# A sport focus of ECEC centres appears especially health-promoting for boys from lower socio-economic background

**DOI:** 10.1093/eurpub/ckac129.582

**Published:** 2022-10-25

**Authors:** A Mayer, R Herr, E Wiedemann, K Diehl, M Blume, S Hoffmann, D Jepsen, L Sundmacher, S Schneider

**Affiliations:** 1 Heidelberg University, Center for Preventive Medicine and Digital Health, Mannheim, Germany; 2 Friedrich-Alexander-Universität, Department of Medical Informatics, Biometry and Epidemiology, Erlangen, Germany; 3 Robert Koch-Institute, Department of Epidemiology and Health Monitoring, Berlin, Germany; 4 Brandenburg University of Technology, Department of Public Health, Senftenberg, Germany; 5 Martin Luther University Halle-Wittenberg, Institute of Medical Sociology, Halle, Germany; 6 Technical University of Munich, Department of Sport and Health Sciences, Munich, Germany

## Abstract

**Background:**

Pediatric overweight is considered one of the 21st century's most serious public health challenges. Many studies investigated individual level determinants of children's body mass index (BMI), yet studies measuring determinants at the meso- level are sparse. As there is a lack of theoretical and empirical knowledge about the role of child care facilities, the aim was to examine the combined effects of family socio-economic position (SEP) and the meso-level variable early childhood education and care (ECEC) centre with sport focus on the BMI of pre-schoolers.

**Methods:**

We used data from the German National Educational Panel Study (NEPS) and included 1,891 children from 224 ECEC centre groups. Multilevel mixed-effects linear regressions were applied to calculate the main association of ECEC centre focus and family SEP, as well as their interaction on children's BMI. All analyses were adjusted for age, migration background, number of siblings, and employment status of parents and were stratified by gender.

**Results:**

Boys attending an ECEC centre with a sport focus have on average a lower BMI than boys from ECEC centres not having this focus. Interactive effects between family SEP and ECEC centre focus were found. Considering predictive margins, boys with low family SEP not attending a sport focused ECEC centre had the highest BMI while boys with low family SEP attending a sport focused ECEC centre had the lowest BMI. For girls, no association regarding ECEC centre focus or interactive effects emerged. Girls in the high family SEP tertile had the lowest BMI in both ECEC centre types.

**Conclusions:**

Our analysis shows the social gradient towards a higher BMI for children from lower SEP families. Considering meso-level factors, we provide evidence for the relevance of ECEC centre characteristics for BMI in boys, whereas for girls the association of family SEP with BMI remains. The ECEC centre focus appears to lower the association of family SEP with BMI for boys.

